# Mayo Adhesive Probability Score Does Not Have Prognostic Ability in Locally Advanced Renal Cell Carcinoma

**DOI:** 10.15586/jkcvhl.v10i1.269

**Published:** 2023-03-21

**Authors:** Benjamin N. Schmeusser, Tad A. Manalo, Yuan Liu, Yash B. Shah, Adil Ali, Manuel Armas-Phan, Dattatraya H. Patil, Reza Nabavizadeh, Kenneth Ogan, Viraj A. Master

**Affiliations:** 1 Department of Urology, Emory University School of Medicine, Atlanta, GA, USA;; 2 Department of Urology, University of Colorado School of Medicine, Denver, CO, USA;; 3 Department of Biostatistics and Bioinformatics, Emory University School of Medicine, Atlanta, GA, USA;; 4 Sidney Kimmel Medical College, Thomas Jefferson University, Philadelphia, PA, USA;; 5 Winship Cancer Institute, Emory University School of Medicine, Atlanta, GA, USA

**Keywords:** body composition, kidney cancer, mayo adhesive probability, renal cell carcinoma, survival

## Abstract

Nephrectomy remains standard treatment for renal cell carcinoma (RCC). The Mayo Adhesive Probability (MAP) score is predictive of adherent perinephric fat and associated surgical complexity, and is determined by assessing perinephric fat and stranding. MAP has additionally predicted progression-free survival (PFS), though primarily reported in stage T1-T2 RCC. Here, we examine MAP’s ability to predict overall survival (OS) and PFS in T3-T4 RCC. From our prospectively maintained RCC database, patients that underwent radical nephrectomy (2009-2016) with available abdominal imaging (<90 days preop) and T3/T4 RCC underwent MAP scoring. Survival analyses were conducted with MAP scores as individual (0-5) and dichotomized (0-3 vs 4-5) using Kaplan-Meier method. Multivariable Cox proportional hazard regression models for PFS and OS were built with backward elimination. 141 patients were included. 134 (95%) and 7 (5%) had pT3 and pT4 disease, respectively. 46.1% of patients had an inferior vena cava thrombus. Mean MAP score was 3.22±1.52, with 75 (53%) patients having a score between 0-3 and 66 (47%) having a score of 4-5. Both male gender (p=0.006) and clear cell histology (p=0.012) were associated with increased MAP scores. On Kaplan-Meier and multivariable analysis, no significant associations were identified between MAP and PFS (HR=1.01, 95% CI 0.85-1.20, p=0.93) or OS (HR=1.01, 95% CI 0.84-1.21, p=0.917). In this cohort of patients with locally advanced RCC, high MAP scores were not predictive of worse PFS or OS.

## Introduction

In 2021, renal cell carcinoma (RCC) was responsible for nearly 14,000 deaths in the United States (1). Diagnosis of RCC has rapidly risen in recent decades, with a doubling in incidence since 1975 (1). Nephrectomy with curative intent remains the gold standard in RCC management; however, image-guided procedures, conservative treatment approaches, and active surveillance have gained popularity. Great interest persists in patient-specific preoperative risk stratification to inform management, rather than relying on postoperative information such as tumor pathology. Specifically, measurements on preoperative imaging may be informative and assist in preoperative prognostication to further guide clinical decision-making.

One radiographic feature that has demonstrated the ability to predict surgical risks and outcomes in RCC is the Mayo Adhesive Probability (MAP) score. MAP estimates the probability of encountering adherent perinephric fat (APF) (2) and has been associated with increased surgical complexity, operative time, and blood loss during partial nephrectomy (PN) (3). Moreover, Thiel et al. explored the association between MAP scores and progression-free survival (PFS). In their analysis, patients with high MAP scores (4–5) experienced inferior PFS (HR = 2.16, 95% CI 1.15–4.06, P = 0.017) following surgery for clinically localized RCC (4).

Accordingly, MAP score appears useful in clinically localized disease and is appealing given its quick and convenient measurement on routine preoperative imaging. However, little is known about its utility in locally advanced RCC. In the study by Thiel et al., 82% of patients had T1–T2 disease. As novel preoperative prognostic factors continue to emerge, understanding their value in all patient populations is necessary. To further elucidate the prognostic utility of MAP, we retrospectively analyzed the associations between preoperative MAP and both PFS and overall survival (OS) in patients with locally advanced nonmetastatic RCC.

## Methods

### Patient selection and data acquisition

Patients that underwent radical nephrectomy (RN) for RCC from 2009 to 2016 were identified in our institutional database. MAP scores were calculated for patients with available computerized tomography (CT) or magnetic resonance imaging (MRI) within 90 days before surgery, as previously described (2). MAP scores were acquired by two Medical Doctorate (MD) candidates pursuing urology residency training and familiar with renal imaging under the direct supervision of an attending urologic oncologist. Patients with T1–T2 disease were excluded. Patient characteristics included race, gender, age of surgery, Eastern Cooperative Oncology Group (ECOG) score, and BMI (<25 or ≥25). Clinical factors including presence of inferior vena cava (IVC) thrombus; laterality of kidney tumor; Fuhrman nuclear grade; presence of necrosis; pathologic N and T stage; stage, size, grade, and necrosis (SSIGN) score; University of California Los Angeles Integrated Staging System (UISS) score; systemic therapy history; corrected calcium; modified Glasgow prognostic score (mGPS); and histology (clear cell [ccRCC] or nonclear cell) were also obtained. All patients provided their informed consent in this study approved by the Institutional Review Board.

## Data analysis

The primary objective of this study was to analyze the prognostic ability of MAP in patients with locally advanced, nonmetastatic RCC. The primary endpoints were PFS and OS.

For survival analyses, MAP scores were analyzed as individual scores (0–5) and dichotomized groups (0–3 vs. 4–5) using the Kaplan–Meier method. In addition, multivariable Cox proportional hazard regression models were built with backward elimination using an alpha level of removal of 0.1. All patient clinicopathologic and demographic features were included in the model. For both PFS and OS, two separate multivariable models were generated to include and exclude SSIGN score, which is only validated in patients with clear cell RCC (ccRCC). Additional subanalyses were conducted in patients with and without presence of IVC tumor thrombus. All statistical tests were two-sided with type I error set at 0.05. Statistical analysis was conducted using SAS Version 9.4 (Cary, NC, USA) and SAS macros developed by the Biostatistics and Bioinformatics Shared Resource at Winship Cancer Institute.

## Results

A summary of patient demographics and clinicopathologic data is represented in Table 1. In total, 141 patients were included, of whom 134 (95%) had pT3 and 7 (5%) had pT4 disease. One hundred and seven (75.9%) patients had clear-cell histology and 65 (46.1%) patients had the presence of an IVC tumor thrombus. The final cohort was primarily male (n = 100, 71%) and white (n = 104; 74%). The median age was 63 years (IQR: 54–72) and median BMI was 28.5 kg/m^2^ (IQR: 24.6–32.6). In total, 47 (33.3%) patients received some form of systemic therapy, all of which were administered postoperatively. Mean MAP score was 3.22 ± 1.52, with 75 (53%) patients having a score between 0–3 and 66 (47%) having a score of 4–5. Both male gender (P = 0.006) and ccRCC histology (P = 0.012) were significantly associated with increased MAP scores, though pathologic staging, ECOG status, and various clinical scoring systems were not. Interestingly, low BMI patients appeared to have lower MAP scores, although the association between these two measures was not significant (P = 0.059).

**Table 1:** Characteristics of study population subdivided by Mayo Adhesive Probability scores.

	MAP score, n (%)							
Covariate	0 (n = 14)	1 (n = 3)	2 (n = 22)	3 (n = 36)	4 (n = 31)	5 (n = 35)	Total (n = 141)	P
Gender								
Male	9 (64.3)	1 (33.3)	14 (63.6)	19 (52.8)	26 (83.9)	31 (88.6)	100 (70.9)	**0.006**
Female	5 (35.7)	2 (66.7)	8 (36.4)	17 (47.2)	5 (16.1)	4 (11.4)	41 (29.1)	
Race								
White	7 (50)	1 (33.3)	15 (68.2)	28 (77.8)	25 (80.6)	28 (80)	104 (73.8)	0.12
Non-white	7 (50)	2 (66.7)	7 (31.8)	8 (22.2)	6 (19.4)	7 (20)	37 (26.2)	
IVC thrombus	5 (35.7)	0 (0)	11 (50)	17 (47.2)	18 (58.1)	14 (40)	65 (46.1)	0.343
ECOG								
≥1	0 (0)	1 (33.3)	5 (22.7)	14 (38.9)	8 (25.8)	5 (14.3)	33 (23.4)	0.052
BMI*	23.9 (20.1–29.0)	30.0 (28.6–31.0)	29.0 (24–35)	26.0 (23.1–30.3)	28.8 (26.2–32.9)	29.9 (27–34.3)	28.50 (24.6–32.6)	0.059
Age*	54.2 (44.2–68.1)	62.7 (51.2–77.7)	59.4 (45.6–71.6)	61.6 (51.5–71.3)	66.9 (55.4–73.4)	63.3 (54.5–73.4)	62.7 (53.8–71.5)	0.212
Nephrectomy side								
Right	8 (57.1)	0 (0)	12 (54.5)	16 (44.4)	17 (54.8)	19 (54.3)	72 (51.1)	0.494
Histology								
ccRCC	6 (42.9)	2 (66.7)	14 (63.6)	32 (88.9)	24 (77.4)	29 (82.9)	107 (75.9)	**0.012**
non-ccRCC	8 (57.1)	1 (33.3)	8 (36.4)	4 (11.1)	7 (22.6)	6 (17.1)	34 (24.1)	
pT stage								
T3	11 (78.6)	3 (100)	22 (100)	35 (97.2)	30 (96.8)	33 (94.3)	134 (95.0)	0.077
T4	3 (21.4)	0 (0)	0 (0)	1 (2.8)	1 (3.2)	2 (5.7)	7 (5.0)	
pN stage								
N1	2 (14.3)	0 (0)	2 (9.1)	4 (11.1)	5 (16.1)	2 (6.3)	15 (10.9)	0.821
Fuhrman nuclear grade								
2	0 (0)	2 (66.7)	4 (18.2)	3 (8.3)	6 (19.4)	5 (14.3)	20 (14.2)	0.246
3	8 (57.1)	1 (33.3)	11 (50)	22 (61.1)	15 (48.4)	16 (45.7)	73 (51.8)	
4	6 (42.9)	0 (0)	7 (31.8)	11 (30.6)	10 (32.3)	14 (40)	48 (34.0)	
Necrosis								
Yes	10 (71.4)	2 (66.7)	17 (77.3)	24 (66.7)	17 (54.8)	24 (68.6)	94 (66.7)	0.659
SSIGN score***								
n (%)	6 (5.6)	2 (1.8)	14 (13.08)	32 (29.9)	24 (22.4)	29 (27.1)	107 (100)	
Mean (std)	7.3 (±1.8)	3.5 (±2.1)	5.1 (±1.6)	6.1 (±1.7)	6.3 (±1.8)	6 (±1.6)	6 (±1.76)	
UISS score**	2.5 (±0.9)	2 (±0.6)	4 (±1.0)	3 (±0.9)	3.5 (±0.9)	2 (± 1.0)	2.94 (±0.93)	0.57
mGPS								
Low	8 (66.7)	2 (100)	8 (38.1)	13 (38.2)	13 (44.8)	12 (35.3)	56 (42.4)	0.211
Intermediate	2 (16.7)	0 (0)	4 (19)	7 (20.6)	9 (31)	14 (41.2)	36 (27.3)	
High	2 (16.7)	0 (0)	9 (42.9)	14 (41.2)	7 (24.1)	8 (23.5)	40 (30.3)	
Missing	–	–	–	–	–	–	9 (6.4%)	
Corrected calcium**	9.8 (±0.6)	9.8 (±0.6)	9.9 (±0.7)	9.8 (±0.8)	9.7 (±0.6)	9.6 (±0.4)	9.74 (±0.65)	0.447
Received systemic therapy	4 (28.6)	0 (0)	6 (27.3)	12 (33.3)	14 (45.2)	11 (31.4)	47 (33.3)	0.547
MAP score								
Mean**	–	–	–	–	–	–	3.22 (±1.52)	
0–3	–	–	–	–	–	–	75 (53.2)	
4–5	–	–	–	–	–	–	66 (46.8)	

MAP, Mayo Adhesive Probability; n, number; IVC, Inferior Vena Cava; ECOG, Eastern Cooperative Oncology Group; BMI, Body mass index; SSIGN, Stage, Size, Necrosis, Grade; ccRCC, Clear-cell renal cell carcinoma; UISS, UCLA Integrated Staging System; mGPS, modified Glasgow prognostic score. *Median (IQR), **Mean (standard deviation[std]), ***non-ccRCC not included (n = 34).

**Figure 1:**
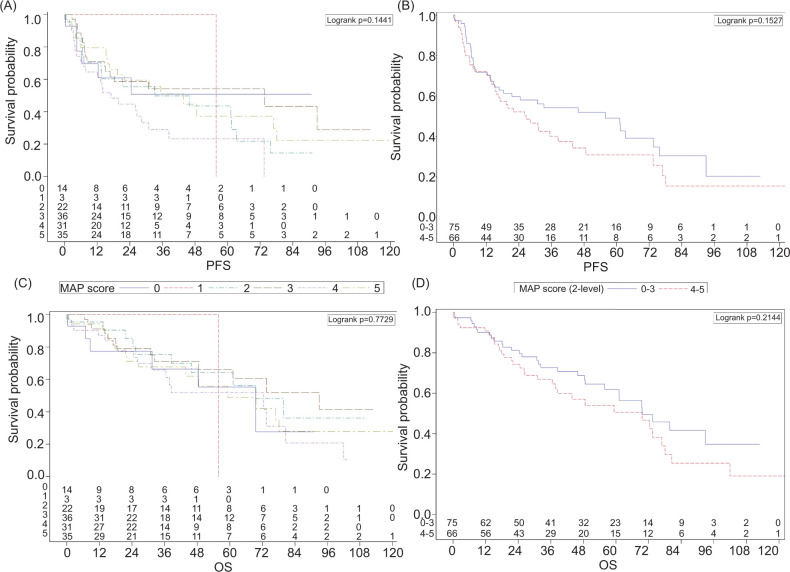
Kaplan–Meier curves for stage T3/T4 renal cell carcinoma patients (n = 141) displaying median progression-free survival (PFS) or overall survival (OS) with either individualized (0–5) or dichotomized (0–3 vs 4–5) Mayo Adhesive Probability (MAP) scores. (A) Median PFS with individualized MAP Scores. (B) Median PFS with dichotomized MAP scores. (C) Median PFS with individualized MAP Scores. (D) Median OS with dichotomized MAP scores.

On Kaplan–Meier analysis, there were no significant associations between continuous or dichotomized MAP scores and PFS or OS (Figure 1). Similarly, multivariable Cox proportional hazard models (Table 2) demonstrated no significant associations both statistically and clinically between MAP and PFS (HR = 1.01, 95% CI 0.85–1.20, P = 0.93) or OS (HR = 1.01, 95% CI 0.84–1.21, P = 0.917). However, no receipt of systemic therapy was associated with better PFS (HR = 0.28, 95% CI 0.18–0.46, P < 0.001). The presence of IVC tumor thrombus was predictive of significantly worse OS (HR = 2.05, 95% CI 1.23–3.39, P = 0.006). These results were similar with and without the inclusion of SSIGN score.

**Table 2:** Multivariable cox proportional hazard model for progression-free survival and overall survival.

Covariate	N	Hazard ratio (95% CI)	P
Progression-free survival*			
MAP score	134	1.01 (0.85–1.20)	0.926
IVC tumor thrombus			
Yes	65	1.55 (0.98–2.47)	0.061
No	76	–	–
Received systemic therapy			
Yes	46	–	–
No	88	0.28 (0.18–0.46)	**<0.001**
Corrected calcium	134	1.38 (0.98–1.94)	0.064
Overall survival**			
MAP score	141	1.01 (0.84–1.21)	0.917
IVC tumor thrombus			
Yes	65	2.05 (1.23–3.39)	**0.006**
No	76	–	–

MAP, Mayo Adhesive Probability; IVC, Inferior vena cava; ECOG, Eastern Cooperative Oncology Group; BMI, Body mass index. *Backward selection with an alpha level of removal of 0.1 was used. The following variables were removed from the model: Age at the Surgery, BMI, clear_cell, ECOG PS at presentation, Gender, Race, and Side of kidney. **Backward selection with an alpha level of removal of 0.1 was used. The following variables were removed from the model: Age at the Surgery, BMI, clear_cell, ECOG PS at presentation, Gender, Race, Ever received systemic therapy, and Side of Kidney.

On subanalysis of patients with the presence of IVC tumor thrombus (Table 3), MAP score continued to have no significant predictive value. Non-white race was associated with worse PFS and no receipt of systemic therapy was associated with improved PFS. For OS, non-white race was additionally associated with worse survival. Notably, only 14 of the patients in the thrombus cases were non-white.

**Table 3:** Multivariable cox proportional hazard model for progression-free survival and overall survival in patients with the presence of an IVC tumor thrombus.

Covariate	N	Hazard ratio (95% CI)	P
Progression-free survival*			
MAP score	64	0.92 (0.67–1.25)	0.589
Non-white race	14	2.38 (1.07–5.33)	**0.034**
Received systemic therapy			
Yes	26	–	–
No	38	0.29 (0.14–0.57)	**<0.001**
Age	64	1.02 (1.00–1.05)	0.082
Corrected calcium	64	1.56 (0.98–2.47)	0.06
Overall survival**			
MAP score	65	1.09 (0.83–1.44)	0.539
Non-white race	14	2.31 (1.10–4.83)	**0.027**

MAP, Mayo Adhesive Probability; IVC, Inferior vena cava; ECOG, Eastern Cooperative Oncology Group; BMI, Body mass index. *Backward selection with an alpha level of removal of 0.1 was used. The following variables were removed from the model: BMI, clear_cell, ECOG PS at presentation, Gender, and Side of kidney. **Backward selection with an alpha level of removal of 0.1 was used. The following variables were removed from the model: Age at the Surgery, BMI, Corrected Calcium, clear_cell, ECOG PS at presentation, Gender, Side of kidney, and systx_ever.

## Discussion

Ultimately, a significant association of MAP with PFS and OS in patients with nonmetastatic T3–T4 RCC was not identified. These findings are important as our understandings of diagnostics, patient-specific prognostication, and the role of body composition in RCC continue to evolve. It is believed that the association between MAP and survival outcomes in RCC exists because perinephric fat thickness and stranding may serve as a proxy for visceral obesity and inflammation (4). Visceral adiposity and inflammation are interconnected, and each has additionally independently been identified as a risk factor for poorer RCC survival outcomes and more aggressive disease (5, 6).

It is unclear why our population of locally advanced RCC patients does not corroborate previous literature demonstrating the prognostic value of MAP scores. It is likely that, for locally advanced disease, extent of disease extension, Tumor, Node, Metastasis (TNM) stage, and other tumor-specific factors play a stronger role in determining survival, thus overpowering the effects of factors such as visceral obesity (7, 8). Moreover, patients with higher visceral obesity may be more likely to present with confounding factors harming survival, including advanced disease or cardiovascular comorbidities.

An important consideration in this cohort of patients with locally advanced disease is the utility of effective systemic therapy (i.e., immune-checkpoint inhibitors [ICI], tyrosine kinase inhibitors) in the neoadjuvant or adjuvant setting. While neoadjuvant therapy prior to nephrectomy has been reported as feasible (9–11), our patient cohort only received adjuvant systemic therapy since neoadjuvant systemic therapy outside of clinical trials is currently not routinely used for nonmetastatic RCC. Patients not receiving any systemic therapy actually experienced better survival, likely as a result of patient selection. Furthermore, in our cohort, there was no difference in receipt of adjuvant systemic therapy by MAP score. Though unable to be captured in this cohort, ICI-induced inflammatory response (12) could potentially be captured by radiographic features measured by MAP scoring given it may partially serve as a proxy for perinephric inflammation (4). Therefore, future studies are warranted in this patient population, specifically.

## Conclusions

This study is the first to examine MAP score in a locally advanced RCC cohort. No significant associations with survival outcomes were identified. Limitations of this study include its retrospective nature and relatively limited sample size. The data enhances our best use of MAP scores to the T1/T2 population. As we move forward in an age of precision medicine and patient-specific risk stratification, a comprehensive understanding of potential prognostic tools is crucial for decision-making and patient counseling.
